# Ginsenoside Rg1 Restores Sirt2/Foxo1 Expression and Alleviates Autism‐Like Behaviors in a Valproic Acid Induced Male Mouse Model

**DOI:** 10.1002/kjm2.70078

**Published:** 2025-07-07

**Authors:** Fan‐Xu Song, Qing‐Wei Wu, Wei Pan, Li‐Jun Liu, Xin Li, Xue Zhou, Zong‐Yao Yu, Xin Ning, Lan‐Min Guo

**Affiliations:** ^1^ Pediatric Health Department, Rehabilitation Medicine College Jiamusi University; Jiamusi University Affiliated Third Hospital; Heilongjiang Province Key Laboratory of Children's Neurorehabilitation Jiamusi Heilongjiang China; ^2^ Traditional Rehabilitation Department, Rehabilitation Medicine College Jiamusi University; Jiamusi University Affiliated Third Hospital; Heilongjiang Province Key Laboratory of Children's Neurorehabilitation Jiamusi Heilongjiang China; ^3^ Medical Administration Department, Rehabilitation Medicine College Jiamusi University; Jiamusi University Affiliated Third Hospital; Heilongjiang Province Key Laboratory of Children's Neurorehabilitation Jiamusi Heilongjiang China; ^4^ Speech Therapy Department, Rehabilitation Medicine College Jiamusi University; Jiamusi University Affiliated Third Hospital; Heilongjiang Province Key Laboratory of Children's Neurorehabilitation Jiamusi Heilongjiang China

**Keywords:** autism spectrum disorder, Ginsenoside Rg1, neuroinflammation and oxidative stress, Sirt2/Foxo1 signaling, valproic acid

## Abstract

This study investigated whether Ginsenoside Rg1 (Rg1) alleviates autism‐like behaviors in mice prenatally exposed to valproic acid (VPA) via Sirt2/Foxo1 signaling. Pregnant C57BL/6J mice received a single intraperitoneal injection of VPA (600 mg/kg) on embryonic Day 12.5 to establish an autism model. At 8 weeks of age, male offspring were randomly divided into four groups: Normal, VPA, VPA + Rg1 (5 mg/kg), and VPA + Rg1 (10 mg/kg). Rg1 was administered once daily for 28 days. Behavioral assessments included grooming, rearing, locomotor activity, social interaction, novel object recognition, open field, and marble‐burying tests. Molecular assays measured Sirt2/Foxo1 expression, inflammatory cytokines, oxidative stress markers in the hippocampus and prefrontal cortex. Nissl staining was performed to evaluate neuronal integrity in the prefrontal cortex and hippocampus. Rg1 administration significantly ameliorated core autism‐like behaviors in VPA‐exposed mice, including deficits in social interaction, recognition memory, and anxiety‐ and compulsive‐like behaviors, as well as excessive grooming and marble‐burying. VPA reduced Sirt2/Foxo1 expression, increased levels of interleukin (IL)‐1β, IL‐6, tumor necrosis factor‐α (TNF‐α), and malondialdehyde (MDA), and decreased superoxide dismutase (SOD) activity in both brain regions. Rg1 treatment reversed these alterations in a dose‐responsive manner, with the 10 mg/kg dose yielding more pronounced behavioral and molecular improvements than the 5 mg/kg dose. Nissl staining revealed significant neuronal loss in VPA‐exposed mice, which was partially restored by Rg1 treatment. These findings suggest that Rg1 alleviates VPA‐induced behavioral and neuropathological abnormalities, potentially via Sirt2/Foxo1‐mediated regulation of neuroinflammation and oxidative stress, and may represent a promising therapeutic strategy for autism spectrum disorder.

## 1 Introduction

Autism spectrum disorder (ASD), originally described by Leo Kanner in 1943 [1], is a neurodevelopmental condition defined by persistent deficits in social communication and interaction, alongside restricted, repetitive behaviors, interests, or activities [2, 3]. A recent systematic review estimated a global median ASD prevalence of 10 per 1000 individuals between 2012 and 2022 [4], with males 4.2 times more frequently affected than females [5]. The multifactorial etiology of ASD encompasses genetic vulnerability and environmental influences—such as maternal infection, obesity, gestational diabetes mellitus, and toxicant exposure—that intersect with critical developmental processes, especially in genetically susceptible individuals [5]. Current ASD management emphasizes early diagnosis, individualized interventions, and a combination of non‐pharmacological and pharmacological strategies [6]. Although risperidone is routinely used for core ASD symptoms and maladaptive behaviors, and methylphenidate for ADHD‐like features, these recommendations are only partially supported by robust evidence [7].

Ginsenoside Rg1 (Rg1), a primary active constituent of ginseng, possesses antiapoptotic, antioxidative, and anti‐inflammatory properties [8]. In several neurological disease models, Rg1 demonstrates neuroprotective efficacy through diverse mechanisms, including spinal cord injury [9], cerebral ischemia/reperfusion injury [10], Parkinson's disease [11], and neurodegenerative disorders such as depression [12], Alzheimer's disease [13, 14]. However, its therapeutic potential in ASD remains unverified. Valproic acid (VPA)—a widely used antiepileptic and mood stabilizer—has teratogenic effects when taken during pregnancy, substantially heightening the offspring's autism risk [2]. Investigations often employ prenatal or postpartum VPA exposure to induce autism‐like behaviors, leveraging embryogenesis as a critical window for neurodevelopmental disruptions. Consequently, VPA treatment is a well‐established model for studying ASD pathogenesis and potential therapeutic interventions [15, 16]. Against this background, we sought to examine whether Rg1 could mitigate VPA‐induced autism‐like behaviors and to elucidate possible molecular mechanisms underlying its action.

## 2 Methods and Materials

### 2.1 Animals and Housing

Pregnant female mice (C57BL/6 strain), aged approximately 8–10 weeks and weighing 20–25 g, were maintained under standard laboratory conditions with a 12 h light/dark cycle, controlled temperature (22°C–24°C), and 40%–60% relative humidity, with food and water provided ad libitum. Vaginal plugs were examined daily, and the detection of a plug was designated as embryonic Day 0.5 (E0.5). All procedures have been approved by the institutional ethics committee, and efforts should be made to minimize any pain or distress.

### 2.2 Prenatal VPA‐Induced Autism Model and Ginsenoside Rg1 Administration

**FIGURE 1:**
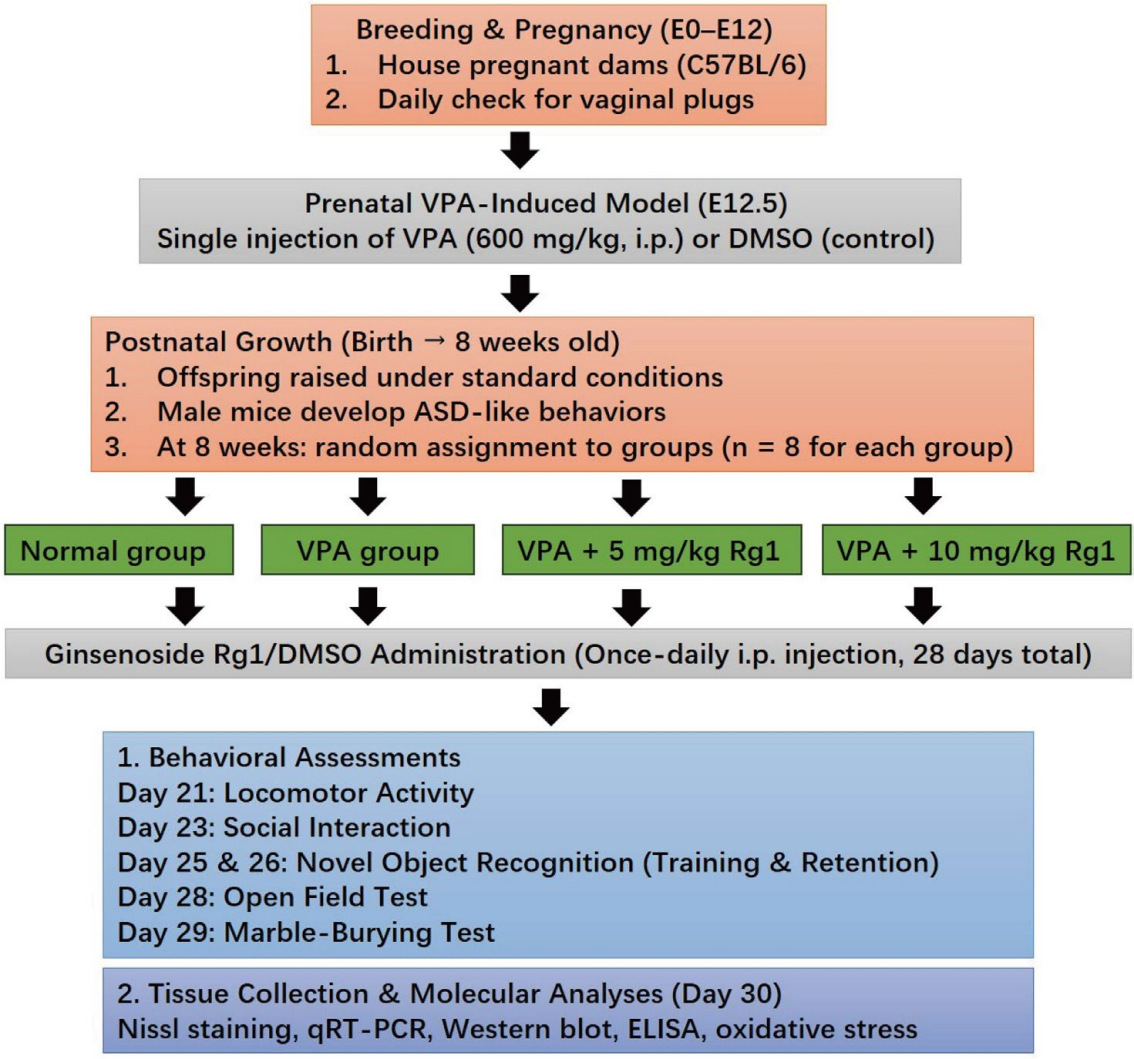
Experimental schedule of the study. Pregnant mice received a single intraperitoneal injection of valproic acid (VPA, 600 mg/kg) or saline on embryonic Day 12.5 (E12.5). Male offspring were weaned and selected at 8 weeks of age. From Day 1 to 28, mice were administered Ginsenoside Rg1 (5 or 10 mg/kg, i.p.) or vehicle once‐daily. Behavioral tests were conducted during the treatment period: Locomotor activity on Day 21, social interaction on Day 23, novel object recognition training and test on Days 25 and 26, open field test on Day 28, and marble‐burying test on Day 29. On Day 30, mice were euthanized for tissue collection.

On embryonic Day 12.5 (E12.5), the dams intended for the autism model received a single intraperitoneal injection of VPA (S3944, Selleck, China) at 600 mg/kg, dissolved in DMSO [17]. Control dams were administered an equivalent volume of saline. Male offspring typically exhibit more pronounced autism‐like behaviors by 4–8 weeks of age [16, 18]. In this study, 8‐week‐old male ASD mice were randomly assigned to two Rg1 treatment groups (5 and 10 mg/kg), each receiving once‐daily intraperitoneal injections for 28 consecutive days [19], or to a vehicle‐only control group. Ginsenoside Rg1 (S3923, Selleck, China) was dissolved in DMSO to ensure a consistent injection volume across groups. Body weight was monitored every 2–3 days throughout the injection period to detect any signs of toxicity or adverse effects. The experimental schedule of the study was illustrated in Figure 1.

### 2.3 Behavioral Assessments

During the final phase of the 28‐day Rg1 (or vehicle) treatment, behavioral evaluations were performed between 10:00 a.m. and 6:00 p.m., with all mice acclimated to the testing environment for at least 1 h before testing [2, 20, 21]. On Day 21, spontaneous locomotor and repetitive behaviors were assessed in a low‐light acrylic chamber (30 × 30 × 30 cm) equipped with infrared sensors. Activity was recorded every 5 min over a 60‐min period. During the final 30 min (31–60 min), grooming frequency and duration were used to quantify repetitive behavior, whereas rearing counts served as an index of exploratory and vertical activity. Total locomotor activity was measured by calculating the cumulative movement over the 30‐min analysis window. On Day 23, social behavior was assessed using the social interaction test. Each mouse was placed in a 25 × 25 × 30 cm open arena with an unfamiliar, age‐ and sex‐matched conspecific for 10 min. The total time engaged in active social behaviors (e.g., sniffing, following) and the total distance moved during interaction was recorded. On Days 25 and 26, cognitive function was evaluated using the novel object recognition (NOR) test. Mice were first habituated to a 30 × 30 × 35 cm test box for 10 min per day over 3 days. On Day 25 (training phase), two different but similarly sized objects were introduced, and exploration time for each object was recorded over 10 min. On Day 26 (retention phase), one familiar object was replaced with a novel one. Total exploration time and the preference index for the novel object (novel object exploration time/total exploration time × 100%) were calculated as indices of recognition memory. On Day 28, anxiety‐like behavior was measured in a dark‐walled square arena (34 × 34 × 24 cm) under vertical illumination. Each mouse was placed in the center and allowed to explore freely for 5 min. Locomotor activity (total distance traveled and average velocity), as well as time spent in the central and peripheral zones, were recorded to assess general activity and anxiety‐related behavior. On Day 29, compulsive‐like behavior was assessed using the marble‐burying test. Mice were placed individually into polypropylene cages (37 × 21 × 14 cm) filled with 5 cm of sawdust bedding, with 20 glass marbles (10 mm) evenly spaced on the surface. After 20 min (without food or water), the number of marbles buried at least two‐thirds was recorded as a measure of repetitive and goal‐directed digging behavior. Total distance moved during the test was also recorded as an index of locomotor activity.

### 2.4 Tissue Collection

Upon completion of behavioral testing (Day 30), mice were deeply anesthetized and transcardially perfused with cold saline to remove circulating blood. Brains were rapidly extracted on ice. The hippocampus and prefrontal cortex were dissected either for paraffin embedding or snap‐frozen in liquid nitrogen and stored at −80°C for subsequent molecular analyses. For histological evaluation, brain tissues were fixed in 4% paraformaldehyde, embedded in paraffin, and sectioned at 5 μm thickness. Coronal sections were stained with 0.1% cresyl violet (Nissl staining) to visualize neuronal cell bodies. Neuronal density was quantified in the CA1 region of the hippocampus and the prefrontal cortex using light microscopy. Neurons with clear nuclei and Nissl substance were counted in standardized fields by blinded investigators.

### 2.5 qRT‐PCR

To quantify mRNA levels of *Sirt2* and *Foxo1*, total RNA was isolated from the hippocampus and prefrontal cortex using TRIzol reagent (Invitrogen, USA), and reverse transcribed into cDNA using the PrimeScript RT Reagent Kit (Takara, Japan). Quantitative PCR amplification was performed with TB Green Premix Ex Taq II (Takara) on a QuantStudio 3 Real‐Time PCR System (Applied Biosystems). Primer sequences were: Sirt2 (F: 5′‐GCCTGGGTTCCCAAAAGGAG; R: 5′‐GAGCGGAAGTCAGGGATACC), Foxo1 (F: 5′‐CCCAGGCCGGAGTTTAACC; R: 5′‐GTTGCTCATAAAGTCGGTGCT), and Gapdh as reference (F: 5′‐AGGTCGGTGTGAACGGATTTG; R: 5′‐TGTAGACCATGTAGTTGAGGTCA). Relative expression was calculated using the 2^−ΔΔCt^ method.

### 2.6 Western Blotting or Protein Analysis

Hippocampal and prefrontal cortex tissues were homogenized in RIPA lysis buffer (Beyotime, China) supplemented with protease and phosphatase inhibitor cocktails. Total protein concentrations were determined using a BCA Protein Assay Kit (Thermo Scientific, USA). Equal amounts of protein (40 μg) were separated by SDS‐PAGE and transferred onto PVDF membranes (Millipore). After blocking in 5% nonfat milk or BSA for 1 h at room temperature, membranes were incubated overnight at 4°C with primary antibodies against SIRT2 and FoxO1. β‐actin was used as an internal control. After washing, membranes were incubated with HRP‐conjugated secondary antibodies and visualized using enhanced chemiluminescence (ECL). Band intensities were quantified using ImageJ software.

### 2.7 Inflammatory Cytokine and Oxidative Stress Assays

Proinflammatory cytokines interleukin‐1β (IL‐1β), interleukin‐6 (IL‐6), and tumor necrosis factor‐α (TNF‐α) in the hippocampus and prefrontal cortex were measured using mouse‐specific ELISA kits from Sigma‐Aldrich: IL‐1β (RAB0275), IL‐6 (RAB0309), and TNF‐α (RAB0477). All assays were performed in duplicate following the manufacturer's instructions. The intra‐ and inter‐assay coefficients of variation were < 10% and < 12%, respectively. Detection sensitivities were 5 pg/mL (IL‐1β), 2 pg/mL (IL‐6), and 60 pg/mL (TNF‐α), with standard curve ranges of 2.74–2000 pg/mL, 0.82–600 pg/mL, and 93.75–6000 pg/mL, respectively. Oxidative stress markers were evaluated by determining malondialdehyde (MDA) levels and superoxide dismutase (SOD) activity. MDA, an indicator of lipid peroxidation, was measured using the Lipid Peroxidation (MDA) Assay Kit (MAK568, Sigma‐Aldrich), whereas SOD activity was assessed using the SOD Assay Kit (MAK528, Sigma‐Aldrich), with a detection range of 0.05–3 U/mL.

### 2.8 Data Analysis

All data were tested for normality using the Shapiro–Wilk test. Results are expressed as mean ± standard deviation (SD). Group comparisons were conducted using one‐way ANOVA followed by Holm‐Sidak's post hoc test, or two‐way ANOVA followed by Tukey's multiple comparisons test, as appropriate. A *p* value less than 0.05 was considered statistically significant.

## 3 Results

### 3.1 Rg1 Improved Locomotor Activity, Rearing, and Grooming in Male Mice Induced by Prenatal VPA Exposure

Body weight was monitored throughout the 29‐day treatment period to evaluate potential toxicity. Two‐way ANOVA revealed significant main effects of treatment and time, but no significant interaction between these factors (*F* = 0.304, *p =* 0.988), indicating similar growth trends across all groups. Although VPA exposure slightly reduced body weight compared to controls from Day 14 onward (all *p <* 0.05), no abnormal weight loss or stagnation was observed in the Rg1 treatment groups. These findings support the conclusion that Rg1 at both 5 and 10 mg/kg doses was well tolerated without signs of systemic toxicity (Figure S1).

**FIGURE 2:**
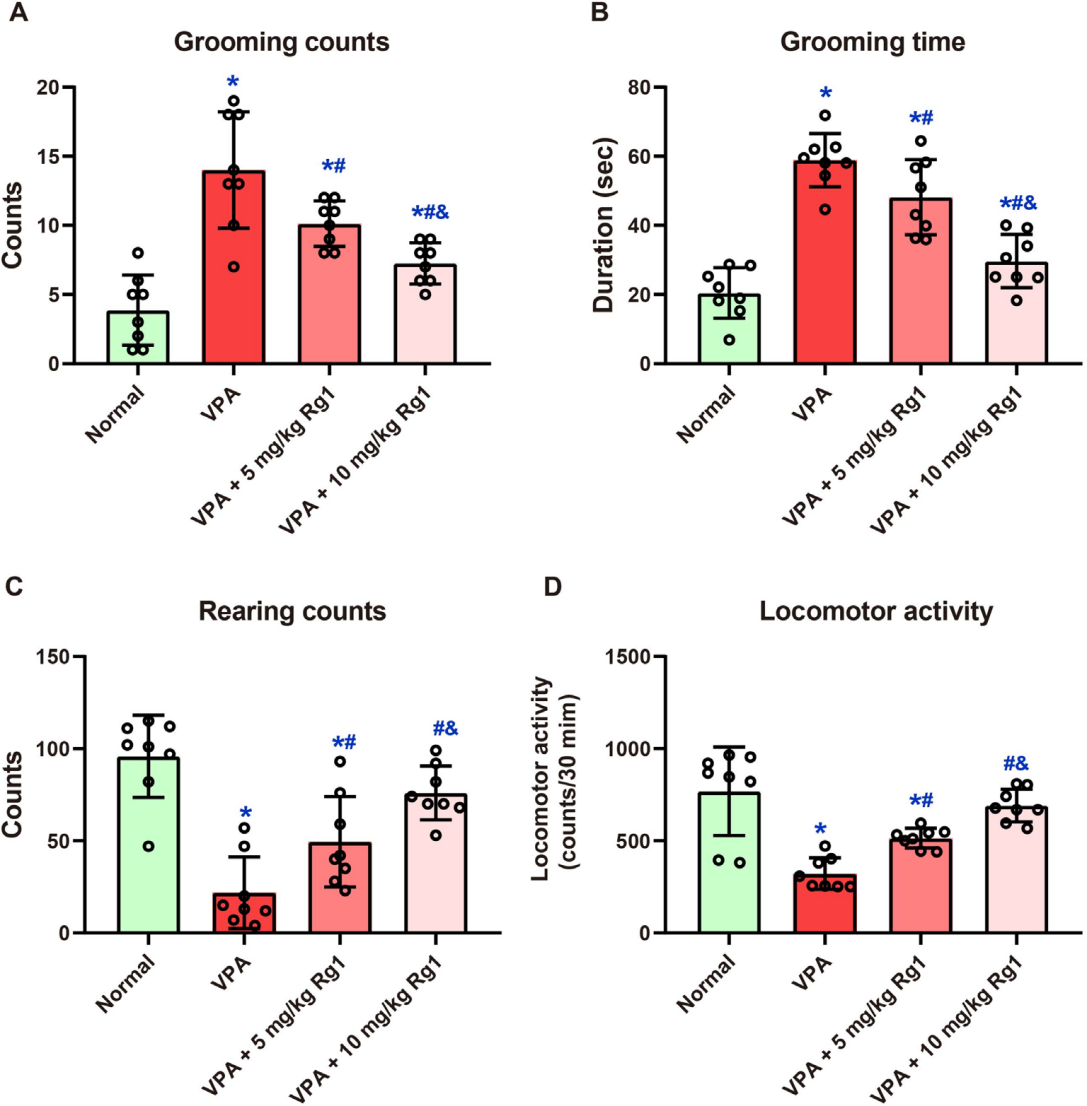
Ginsenoside Rg1 mitigates VPA‐induced alterations in grooming, rearing, and locomotor behavior in mice. On Day 21, behavioral parameters were evaluated during the second half (31–60 min) of a 60‐min observation period. Repetitive behavior was measured by analyzing grooming frequency (A) and grooming duration (B), whereas locomotor and vertical exploratory activity were assessed via total locomotion (C) and rearing events (D), respectively. Data are expressed as mean ± SD (*n* = 8 per group). Statistical analysis was performed using one‐way ANOVA with Holm‐Sidak's post hoc test. *, #, and & indicate *p <* 0.05 compared with the Normal group, VPA group, and VPA + 5 mg/kg Rg1 group, respectively.

On Day 21, one‐way ANOVA revealed a significant effect among groups in the grooming behavior test (*F* (3, 28) = 20.37, *p <* 0.001, Figure 2A). Mice in the VPA group exhibited significantly increased grooming counts (14.00 ± 4.21) compared to the Normal group (3.88 ± 2.53, *p <* 0.001). Rg1 treatment reduced this behavior at both doses: grooming counts were 10.13 ± 1.64 in the VPA + 5 mg/kg group (*p =* 0.023 vs. VPA) and 7.25 ± 1.49 in the VPA + 10 mg/kg group (*p <* 0.001 vs. VPA). In the grooming time analysis, one‐way ANOVA revealed a significant main effect of treatment (*F* (3, 28) = 33.42, *p <* 0.001, Figure 2B). The VPA group displayed significantly prolonged grooming duration (58.88 ± 7.71 s) compared to the Normal group (20.46 ± 7.31 s, *p <* 0.001). Administration of Rg1 reduced grooming time: 48.18 ± 10.88 s in the VPA + 5 mg/kg group (*p =* 0.036 vs. VPA) and 29.65 ± 7.69 s in the VPA + 10 mg/kg group (*p <* 0.001 vs. VPA). In the analysis of rearing counts, one‐way ANOVA revealed a significant effect of treatment (*F* (3, 28) = 19.59, *p <* 0.001, Figure 2C). The VPA group exhibited markedly reduced rearing behavior (21.88 ± 19.39) compared to the Normal group (95.88 ± 22.36, *p <* 0.001). Rg1 administration improved rearing counts to 49.5 ± 24.51 (5 mg/kg, *p =* 0.036) and 76.0 ± 14.59 (10 mg/kg, *p <* 0.001). In the 30‐min locomotor activity test, one‐way ANOVA showed a significant group effect (*F* (3, 28) = 16.76, *p <* 0.001, Figure 2D). VPA exposure significantly reduced locomotor activity (322.4 ± 85.47) compared to the Normal group (769.3 ± 240.3, *p <* 0.001). Rg1 treatment restored activity levels to 514.8 ± 52.75 (*p =* 0.004 vs. VPA) and 691.9 ± 88.3 (*p <* 0.001 vs. VPA), with the higher dose resulting in greater recovery.

### 3.2 Ginsenoside Rg1 Improved Social Interaction and Recognition Memory in Male Mice Following Prenatal VPA Exposure

**FIGURE 3:**
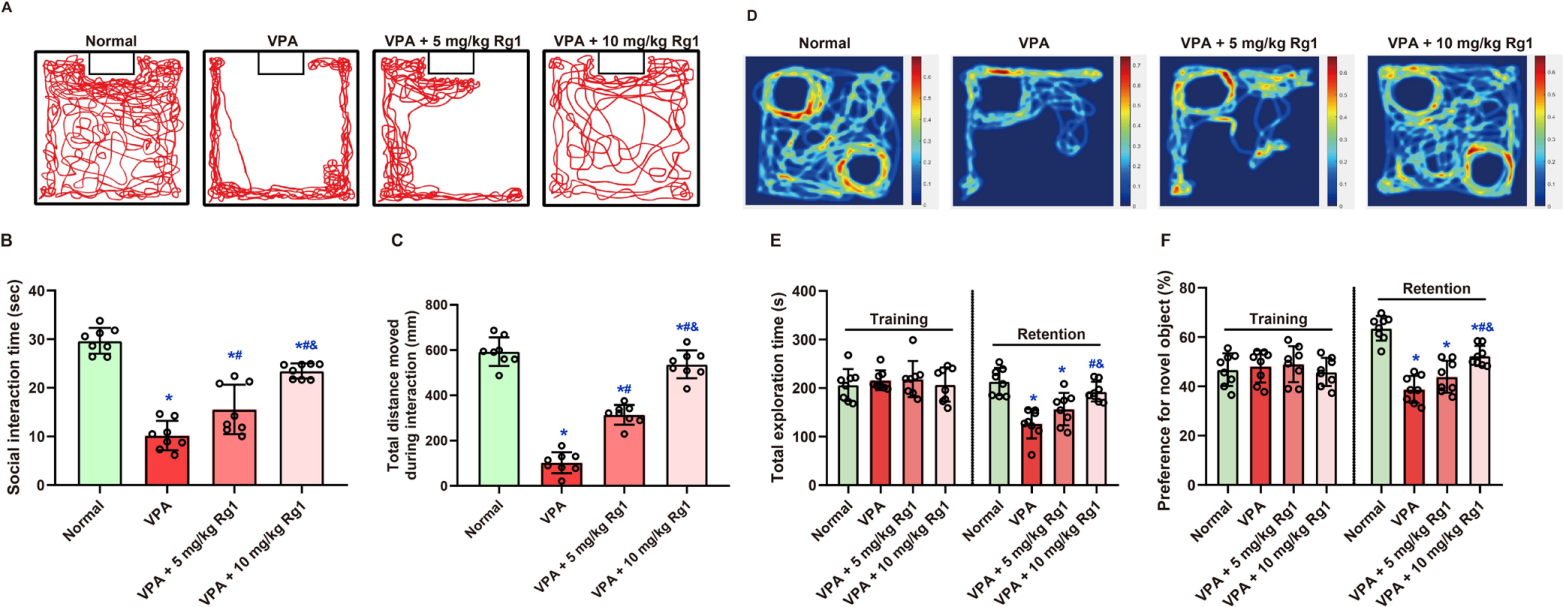
Ginsenoside Rg1 improved social interaction and recognition memory impairments in male mice following prenatal VPA exposure. (A) Representative movement traces during the social interaction test on Day 23. (B, C) Quantification of interaction time (B) and total movement distance (C) during the social interaction test. (D) Representative heatmaps from the novel object recognition (NOR) test on Day 26. (E, F) Quantification of total object exploration time (E) and percentage preference for the novel object (F) during the training and retention phases. Data are expressed as mean ± SD (*n* = 8 per group). Statistical significance was determined by one‐way ANOVA followed by Holm‐Sidak's post hoc test. *, #, and & indicate *p <* 0.05 compared with the Normal group, VPA group, and VPA + 5 mg/kg Rg1 group, respectively.

On Day 23, social behavior was evaluated using the social interaction test (Figure 3A). Social interaction behavior differed significantly among groups, both in terms of interaction times (*F* (3, 28) = 53.02, *p <* 0.001, Figure 3B) and total movement distance (*F* (3, 28) = 136.9, *p <* 0.001, Figure 3C). VPA‐exposed mice exhibited markedly reduced interaction times (10.18 ± 3.04 s) and significantly decreased total movement distances (102.2 ± 46.3 mm) compared to Normal controls (interaction time: 29.66 ± 2.65 s; total distance: 593.5 ± 62.99 mm, both *p <* 0.001). Treatment with Rg1 significantly ameliorated these deficits in a dose‐responsive manner. The 5 mg/kg Rg1 group showed partial improvements in interaction times (15.57 ± 5.08 s, *p =* 0.003 vs. VPA) and total movement distances (314.2 ± 43.52 mm, *p <* 0.001 vs. VPA), whereas the 10 mg/kg Rg1 group exhibited more pronounced improvements in both interaction times (23.47 ± 1.56 s, *p <* 0.001 vs. VPA) and total movement distances (537.2 ± 61.73 mm, *p <* 0.001 vs. VPA), approaching the performance of Normal controls (interaction time, *p =* 0.002 vs. Normal; total distance, *p =* 0.048 vs. Normal).

Cognitive performance was further assessed through the NOR test (Figure 3D), measuring total exploration times during both training and retention phases (Figure 3E), alongside preferences for novel objects (Figure 3F). No significant differences were found in total exploration time during the training phase across groups (all *p* > 0.05). However, one‐way ANOVA revealed a significant group effect on total exploration time during the retention phase (*F* (3, 28) = 14.76, *p <* 0.001). Mice in the VPA group showed markedly reduced exploration (126.4 ± 30.25 s) compared to the Normal group (213.5 ± 27.91 s, *p <* 0.001), suggesting impaired recognition memory. Rg1 treatment partially reversed this impairment: the VPA + 5 mg/kg group increased to 156.7 ± 33.50 s (*p =* 0.082 vs. VPA), and the VPA + 10 mg/kg group further improved to 192.8 ± 20.32 s (*p <* 0.001 vs. VPA; *p =* 0.156 vs. Normal), with a significant difference between Rg1 doses (*p =* 0.050). Similarly, for novel object preference during the retention phase, one‐way ANOVA revealed a significant treatment effect (*F* (3, 28) = 33.14, *p <* 0.001). The VPA group exhibited a substantial reduction in preference (38.75% ± 5.47%) compared to the Normal group (63.56% ± 4.98%, *p <* 0.001). Rg1 treatment improved recognition: 43.90% ± 6.50% in the VPA + 5 mg/kg group (*p =* 0.064 vs. VPA) and 52.34% ± 4.07% in the VPA + 10 mg/kg group (*p <* 0.001 vs. VPA), with a significant difference between Rg1 doses (*p =* 0.007). These findings suggest that Ginsenoside Rg1 mitigates VPA‐induced cognitive deficits in a dose‐responsive manner.

### 3.3 Ginsenoside Rg1 Alleviated Anxiety‐Like and Compulsive‐Like Behaviors in Male Mice Following Prenatal VPA Exposure

**FIGURE 4:**
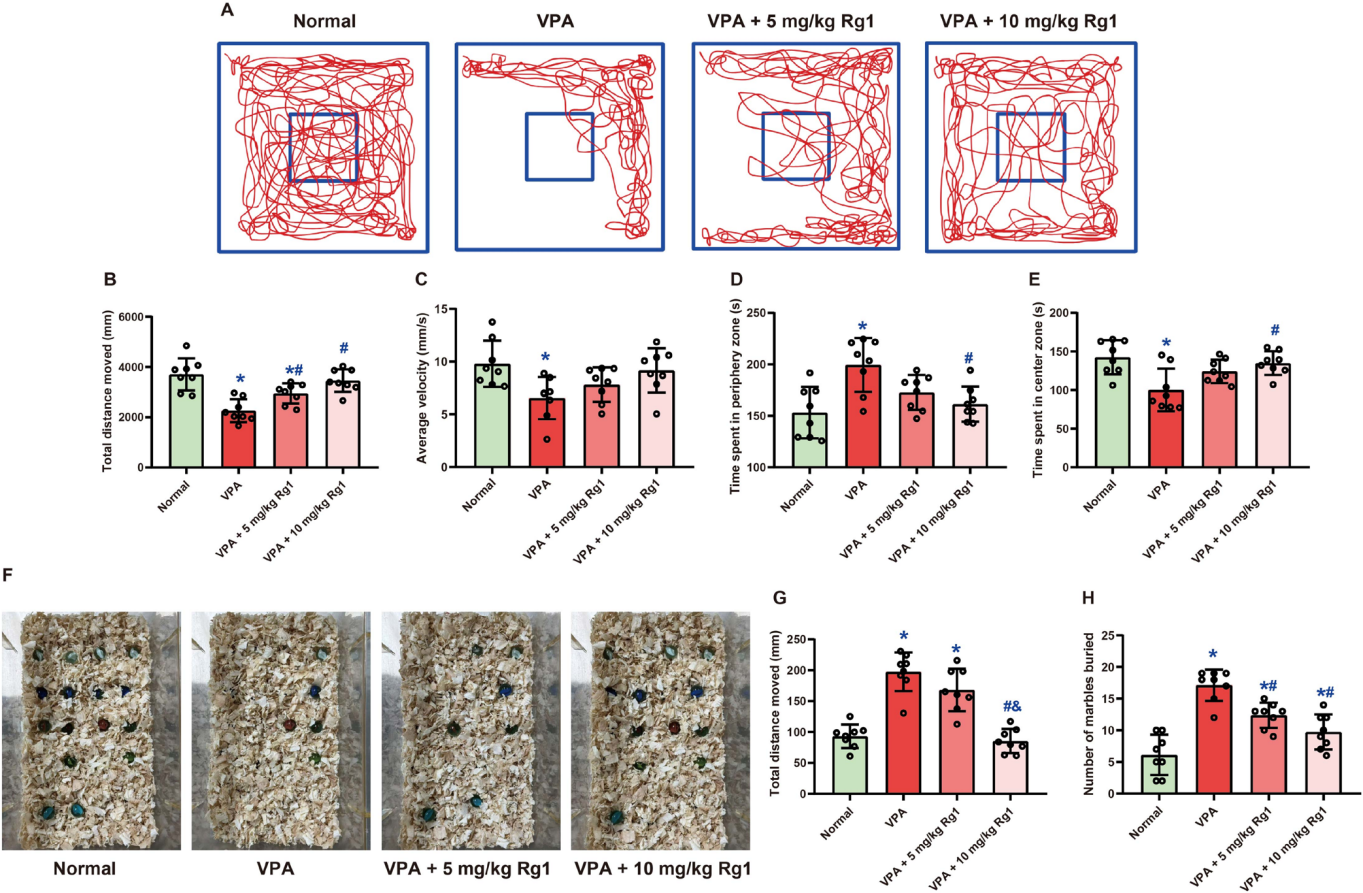
Ginsenoside Rg1 alleviated anxiety‐like and compulsive‐like behaviors in male mice following prenatal VPA exposure. (A) Representative tracking images from the open field test on Day 28. (B–E) Quantification of total distance moved (B), average velocity (C), time spent in the periphery (D), and time spent in the center zone (E) during the open field test. (F) Representative images from the marble‐burying test on Day 29. (G, H) Quantification of total distance moved (G) and the number of marbles buried (H), reflecting locomotor activity and compulsive‐like behavior, respectively. Data are expressed as mean ± SD (*n* = 8 per group). Statistical significance was determined by one‐way ANOVA followed by Holm‐Sidak's post hoc test. *, #, and & indicate *p <* 0.05 compared with the Normal group, VPA group, and VPA + 5 mg/kg Rg1 group, respectively.

On Day 28, anxiety‐like behavior was assessed using the open field test (Figure 4A–E). One‐way ANOVA revealed a significant group effect on total distance moved (*F* (3, 28) = 13.32, *p <* 0.001, Figure 4B). Mice in the VPA group showed markedly reduced locomotor activity (2263 ± 455.7 mm) compared to the Normal group (3710 ± 639.7 mm, *p <* 0.001), indicating enhanced anxiety‐like behavior. Rg1 treatment partially restored activity levels: the 5 mg/kg group increased to 2949 ± 403.9 mm (*p =* 0.029 vs. VPA), whereas the 10 mg/kg group further improved to 3461 ± 448.9 mm (*p <* 0.001 vs. VPA; *p =* 0.324 vs. Normal). No significant difference was found between the two Rg1 doses (*p =* 0.094). Similarly, average velocity differed significantly among groups (*F* (3, 28) = 4.219, *p =* 0.014, Figure 4C). VPA‐treated mice showed reduced average velocity (6.549 ± 1.999 mm/s) compared to Normal controls (9.791 ± 2.191 mm/s, *p =* 0.018). Rg1 treatment increased velocity to 7.819 ± 1.635 mm/s (5 mg/kg) and 9.169 ± 2.095 mm/s (10 mg/kg), but these increases were not statistically significant (*p =* 0.461 and 0.067 vs. VPA, respectively). For spatial preference, one‐way ANOVA revealed a significant effect on time spent in the periphery zone (*F* (3, 28) = 6.914, *p =* 0.001, Figure 4D). VPA‐exposed mice spent more time in the periphery (199.5 ± 26.12 s) than Normal mice (153.2 ± 24.98 s, *p =* 0.001), suggesting increased anxiety. Rg1 treatment reduced periphery time to 172.7 ± 16.94 s (5 mg/kg, *p =* 0.078 vs. VPA) and 161.4 ± 17.05 s (10 mg/kg, *p =* 0.008 vs. VPA). Only the higher dose significantly reversed the abnormality, with no significant difference between the two doses (*p =* 0.518). Conversely, time spent in the center zone—an anxiolytic indicator—also showed a significant group effect (*F* (3, 28) = 6.37, *p =* 0.002, Figure 4E). VPA mice exhibited reduced center time (100.3 ± 27.53 s) relative to Normal mice (142.5 ± 21.98 s, *p =* 0.002). Rg1 treatment elevated center time to 124.2 ± 15.11 s (5 mg/kg, *p =* 0.107 vs. VPA) and 134.9 ± 15.37 s (10 mg/kg, *p =* 0.011 vs. VPA), with the latter dose significantly alleviating VPA‐induced anxiety‐like behavior (*p =* 0.521 between Rg1 groups).

On Day 29, behavioral alterations were assessed using the marble‐burying test, which included analysis of total distance moved and the number of marbles buried (Figure 4F–H). One‐way ANOVA revealed a significant group effect on locomotor activity (*F* (3, 28) = 33.84, *p <* 0.001, Figure 4G). Mice in the VPA group exhibited significantly elevated total distance moved (197.4 ± 31.26 m) compared to the Normal group (93.13 ± 19.01 m, *p <* 0.001), which was attenuated by the Rg1 treatment. Mice treated with 5 mg/kg Rg1 showed a partial reduction (168.0 ± 34.39 m, *p =* 0.074 vs. VPA), whereas those receiving 10 mg/kg Rg1 exhibited significant behavioral normalization (85.33 ± 19.48 m, *p <* 0.001 vs. VPA; *p =* 0.567 vs. Normal). A significant difference between the two Rg1 doses (*p <* 0.001) further supported a dose‐dependent effect. Compulsive‐like behavior, reflected by the number of marbles buried, also showed a significant group effect (*F* (3, 28) = 24.59, *p <* 0.001, Figure 4H). VPA‐exposed mice buried significantly more marbles (17.13 ± 2.475) than the Normal controls (6.13 ± 3.182, *p <* 0.001), indicating excessive repetitive digging. Treatment with Ginsenoside Rg1 significantly reduced this behavior: the 5 mg/kg group buried 12.38 ± 1.996 marbles (*p =* 0.004 vs. VPA), and the 10 mg/kg group buried 9.75 ± 2.765 marbles (*p <* 0.001 vs. VPA; *p =* 0.021 vs. Normal). Although a trend toward difference was observed between the two Rg1 doses (*p =* 0.057), it did not reach statistical significance. Overall, these findings demonstrate that Rg1 effectively mitigates VPA‐induced hyperactivity and compulsive‐like behaviors, particularly at higher doses.

### 3.4 Ginsenoside Rg1 Restored Sirt2/Foxo1 Expression and Mitigated VPA‐Induced Neuronal Loss in the Prefrontal Cortex and Hippocampus

**FIGURE 5:**
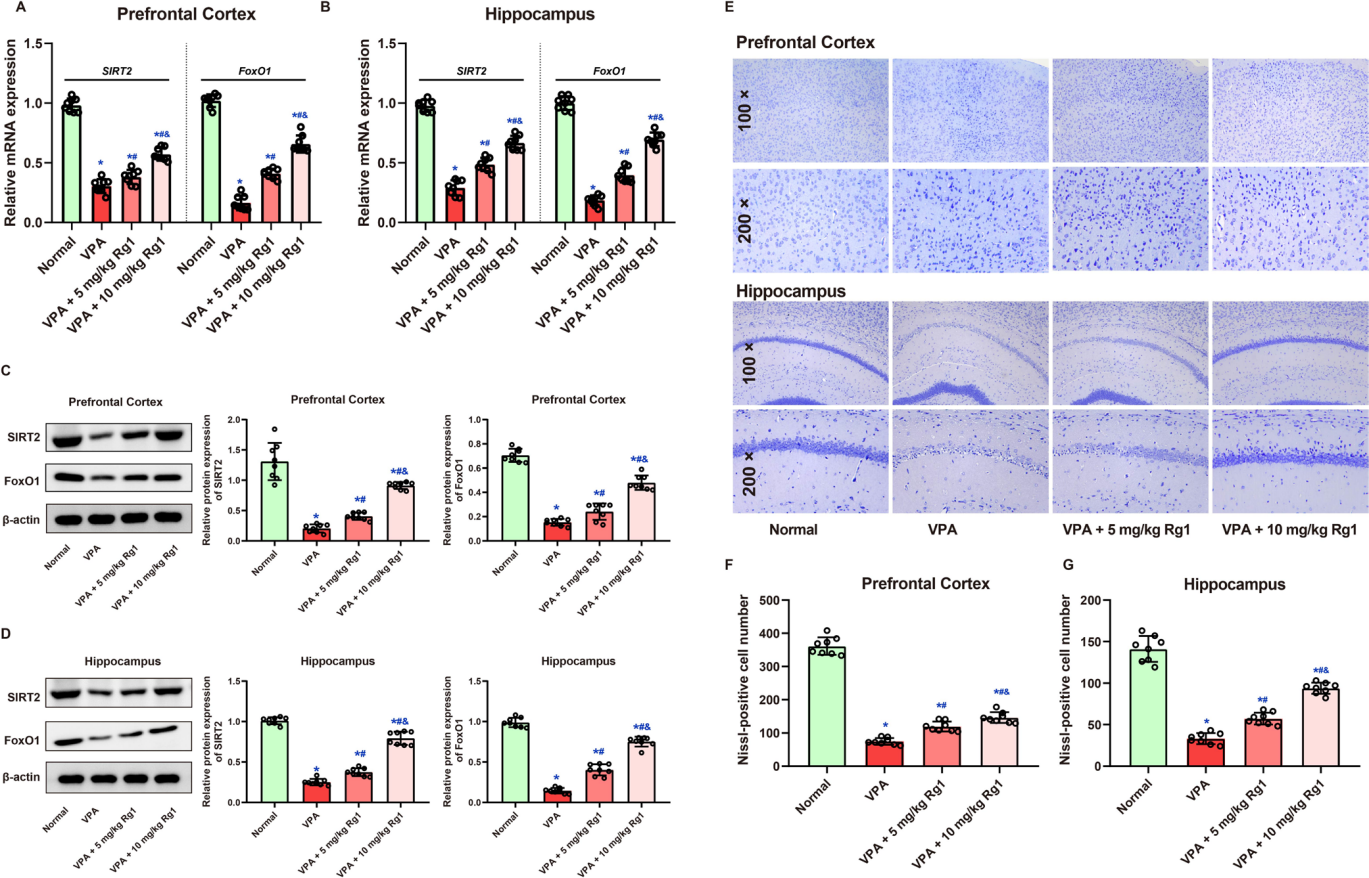
Ginsenoside Rg1 restored Sirt2/Foxo1 expression and mitigated VPA‐induced neuronal loss in the prefrontal cortex and hippocampus. (A, B) mRNA expression levels of Sirt2 (A) and Foxo1 (B) in the prefrontal cortex and hippocampus. (C, D) Representative Western blots and quantitative analysis of Sirt2 and Foxo1 protein expression. (E) Representative images of Nissl‐stained coronal brain sections showing neuronal morphology in the prefrontal cortex and hippocampus. (F, G) Quantification of neuronal numbers in the prefrontal cortex (F) and hippocampus (G). Data are shown as mean ± SD (*n* = 8 per group). Statistical analysis was conducted using one‐way ANOVA with Holm‐Sidak's post hoc test. *, #, and & indicate *p <* 0.05 compared with the Normal group, VPA group, and VPA + 5 mg/kg Rg1 group, respectively.

Molecular analysis (Figure 5A–D) showed that both Sirt2 and Foxo1 mRNA and protein expression levels were significantly reduced in the hippocampus and prefrontal cortex of VPA‐treated mice compared to the Normal group (*p <* 0.05). Treatment with 5 mg/kg Rg1 resulted in a partial recovery of Sirt2/Foxo1 expression, whereas 10 mg/kg Rg1 led to a more substantial restoration of both Sirt2 protein levels and Foxo1 signaling (*p <* 0.05). These findings suggest that higher dose Rg1 may exert stronger neuroprotective effects by enhancing Sirt2 expression and regulating Foxo1 activity, thereby contributing to improved synaptic plasticity and behavioral outcomes in VPA‐induced autism‐like phenotypes. Nissl staining revealed significant VPA‐induced neuronal loss in both the prefrontal cortex and hippocampus, which was ameliorated by Ginsenoside Rg1 treatment in a dose‐related manner (Figure 5E). One‐way ANOVA showed a significant group effect on neuronal number in the prefrontal cortex (*F* (3, 28) = 402.8, *p <* 0.001, Figure 5F). Mice exposed to VPA exhibited markedly reduced neuronal counts (74.63 ± 10.23) compared to the Normal group (361.5 ± 26.37, *p <* 0.001). Ginsenoside Rg1 treatment increased neuronal survival, with the 5 mg/kg group showing 119.5 ± 15.03 neurons (*p <* 0.001 vs. VPA) and the 10 mg/kg group showing 146.4 ± 16.28 neurons (*p <* 0.001 vs. VPA). A significant difference was also found between the two Rg1‐treated groups (*p =* 0.006), indicating improved efficacy at the higher dose. Nonetheless, neuron counts in both treatment groups remained significantly lower than those in the Normal group (*p <* 0.001), indicating partial rescue. In the hippocampus, a significant group effect was also observed (*F* (3, 28) = 183.3, *p <* 0.001, Figure 5G). VPA‐exposed mice showed a substantial reduction in neuronal numbers (33.25 ± 6.63) compared to controls (141.1 ± 15.62, *p <* 0.001). Ginsenoside Rg1 administration led to a clear recovery of neuron numbers: 57.25 ± 7.23 at 5 mg/kg (*p <* 0.001 vs. VPA) and 93.88 ± 6.75 at 10 mg/kg (*p <* 0.001 vs. VPA), with a significant difference between the two doses (*p <* 0.001). These findings suggest that Rg1 improves neuronal survival after VPA exposure, with greater benefit at the higher dose.

### 3.5 Ginsenoside Rg1 Attenuated VPA‐Induced Inflammation and Oxidative Stress in the Hippocampus and Prefrontal Cortex

As shown in Table 1, one‐way ANOVA revealed significant group effects on all inflammatory and oxidative stress markers in the hippocampus (all *p <* 0.001): IL‐1β (*F* (3, 28) = 141.3), IL‐6 (*F* (3, 28) = 120.8), TNF‐α (*F* (3, 28) = 104.7), MDA (*F* (3, 28) = 54.79), and SOD (*F* (3, 28) = 66.98). Compared to the Normal group (IL‐1β: 35.36 ± 2.45 pg/mg; IL‐6: 30.70 ± 2.90 pg/mg; TNF‐α: 23.13 ± 1.79 pg/mg; MDA: 1.37 ± 0.14 nmol/mg; SOD: 8.80 ± 0.64 U/mg), the VPA group exhibited significantly elevated IL‐1β (69.76 ± 5.40 pg/mg), IL‐6 (55.56 ± 2.25 pg/mg), TNF‐α (44.85 ± 3.48 pg/mg), and MDA (2.13 ± 0.14 nmol/mg), alongside reduced SOD (5.05 ± 0.50 U/mg) (all *p <* 0.05). Rg1 treatment ameliorated these abnormalities. The 5 mg/kg Rg1 group showed partial recovery (IL‐1β: 50.26 ± 2.84; IL‐6: 38.99 ± 2.84; TNF‐α: 30.04 ± 2.35; MDA: 1.66 ± 0.08; SOD: 6.97 ± 0.59, all *p <* 0.05), whereas the 10 mg/kg group showed further improvement (IL‐1β: 40.61 ± 2.97; IL‐6: 33.66 ± 3.34; TNF‐α: 25.89 ± 2.79; MDA: 1.52 ± 0.14; SOD: 7.84 ± 0.46, all *p <* 0.05), with all parameters significantly different from the VPA group and closer to Normal levels.

**TABLE 1:** Ginsenoside Rg1 attenuated VPA‐induced inflammation and oxidative stress in the hippocampus and prefrontal cortex.

	Normal	VPA	VPA + 5 mg/kg Rg1	VPA + 10 mg/kg Rg1	*F* (3, 28)	*p*
Hippocampus						
IL‐1β (pg/mg)	35.36 ± 2.45	69.76 ± 5.40*	50.26 ± 2.84*^#^	40.61 ± 2.97*^#&^	141.3	< 0.001
IL‐6 (pg/mg)	30.70 ± 2.90	55.56 ± 2.25*	38.99 ± 2.84*^#^	33.66 ± 3.34*^#&^	120.8	< 0.001
TNF‐α (pg/mg)	23.13 ± 1.79	44.85 ± 3.48*	30.04 ± 2.35*^#^	25.89 ± 2.79*^#&^	104.7	< 0.001
MDA (nmol/mg)	1.37 ± 0.14	2.13 ± 0.14*	1.66 ± 0.08*^#^	1.52 ± 0.14*^#&^	54.79	< 0.001
SOD (U/mg)	8.80 ± 0.64	5.05 ± 0.50*	6.97 ± 0.59*^#^	7.84 ± 0.46*^#&^	66.98	< 0.001
Prefrontal cortex						
IL‐1β (pg/mg)	28.81 ± 1.80	60.63 ± 2.99*	42.11 ± 3.51*^#^	34.49 ± 3.12*^#&^	179.6	< 0.001
IL‐6 (pg/mg)	23.48 ± 2.34	50.11 ± 2.78*	32.28 ± 3.27*^#^	27.23 ± 3.07*^#&^	133.5	< 0.001
TNF‐α (pg/mg)	17.38 ± 2.45	40.15 ± 3.08*	25.86 ± 3.16*^#^	21.73 ± 2.54*^#&^	97.64	< 0.001
MDA (nmol/mg)	1.13 ± 0.10	1.99 ± 0.14*	1.56 ± 0.12*^#^	1.37 ± 0.14*^#&^	69.73	< 0.001
SOD (U/mg)	8.08 ± 0.57	4.03 ± 0.68*	6.17 ± 0.6*^#^	6.8 ± 0.52*^#&^	64.98	< 0.001

*Note*: Inflammatory cytokines including interleukin‐1β (IL‐1β), interleukin‐6 (IL‐6), and tumor necrosis factor‐α (TNF‐α) were quantified using commercially available mouse ELISA kits. Oxidative stress was evaluated by measuring malondialdehyde (MDA) levels and superoxide dismutase (SOD) activity. Data are shown as mean ± SD (*n* = 8 per group). Statistical analysis was conducted using one‐way ANOVA with Holm‐Sidak's post hoc test. *, #, and & indicate *p* < 0.05 compared with the Normal group, VPA group, and VPA + 5 mg/kg Rg1 group, respectively.

In the prefrontal cortex, similar significant effects were observed (all *p <* 0.001): IL‐1β (*F* (3, 28) = 179.6), IL‐6 (*F* (3, 28) = 133.5), TNF‐α (*F* (3, 28) = 97.64), MDA (*F* (3, 28) = 69.73), and SOD (*F* (3, 28) = 64.98). The VPA group showed marked increases in IL‐1β (60.63 ± 2.99), IL‐6 (50.11 ± 2.78), TNF‐α (40.15 ± 3.08), and MDA (1.99 ± 0.14), with reduced SOD levels (4.03 ± 0.68) compared to Normal controls (IL‐1β: 28.81 ± 1.80; IL‐6: 23.48 ± 2.34; TNF‐α: 17.38 ± 2.45; MDA: 1.13 ± 0.10; SOD: 8.08 ± 0.57, all *p <* 0.05). Ginsenoside Rg1 reversed these changes in a dose‐responsive manner. The 5 mg/kg group exhibited moderate improvement (IL‐1β: 42.11 ± 3.51; IL‐6: 32.28 ± 3.27; TNF‐α: 25.86 ± 3.16; MDA: 1.56 ± 0.12; SOD: 6.17 ± 0.60, all *p <* 0.05), and the 10 mg/kg group showed greater restoration (IL‐1β: 34.49 ± 3.12; IL‐6: 27.23 ± 3.07; TNF‐α: 21.73 ± 2.54; MDA: 1.37 ± 0.14; SOD: 6.80 ± 0.52, all *p <* 0.05).

## 4 Discussion

Our findings showed that VPA exposure caused significant deficits in spontaneous locomotor activity and exploratory drive, as well as elevated grooming behavior. These changes are consistent with previous reports that prenatal VPA impairs motor function and increases repetitive actions akin to ASD [22, 23]. Rg1 administration, especially at 10 mg/kg, effectively reversed these impairments, suggesting a role in modulating neural circuits underlying both motor coordination and repetitive behavior. The attenuation of excessive grooming could relate to Rg1's influence on serotonin or dopaminergic pathways [11, 24, 25], since repetitive behaviors in ASD‐like models have been linked to disrupted monoaminergic signaling [26]. Moreover, the restored rearing behavior implies that Rg1 may enhance basal ganglia and cortical interactions crucial for goal‐directed exploration.

VPA‐exposed mice also displayed marked deficits in social engagement and NOR, aligning with the core ASD symptoms of social communication deficits and restricted interests [2]. Rg1 treatment rescued these behaviors in a dose‐responsive manner, indicating that its neuroprotective scope extends beyond mere motor improvements to encompass higher‐order cognitive and social processes. The hippocampus, notably vulnerable to oxidative and inflammatory insults induced by VPA, is central for memory formation, whereas the prefrontal cortex orchestrates social behavior and executive functions [27]. By mitigating inflammation and modulating key molecular pathways in these regions, Rg1 may restore synaptic plasticity and neuronal connectivity that underlie social cognition and memory consolidation. Our open field and marble‐burying results demonstrated that prenatal VPA exposure induced significant anxiety‐like and compulsive behavioral alterations in mice. Specifically, VPA‐exposed mice spent more time in the periphery of the open field, a well‐established indicator of heightened anxiety. Concurrently, these mice exhibited increased marble‐burying behavior, reflecting elevated repetitive and perseverative tendencies commonly associated with ASD. Although grooming behavior was also exaggerated in our model, the enhanced marble‐burying suggests a distinct compulsive‐like phenotype, possibly linked to aberrant functioning of corticostriatal and limbic circuits. Rg1 administration—particularly at the higher dose—effectively attenuated both the anxiety‐like phenotype and excessive repetitive digging. This behavioral recovery is likely mediated by Rg1's well‐documented anti‐inflammatory and antioxidative properties, which have been shown to modulate neuroimmune responses and synaptic plasticity. Together, these findings reinforce the therapeutic potential of Rg1 in alleviating ASD‐related behavioral impairments by targeting underlying neurobiological mechanisms.

On a molecular level, VPA downregulated Sirt2 and Foxo1 in both the prefrontal cortex and hippocampus. Sirt2 can regulate cytoplasmic deacetylation processes that influence neuroinflammation [28], whereas Foxo1 has been implicated in neuronal survival and synaptic plasticity by governing antioxidant responses and energy metabolism [29]. Guo et al. demonstrated that Sirt2 gene deletion in an ASD mouse model led to enhanced acetylation of Foxo1, excessive autophagy, and aggravated neuroinflammation in the hippocampus, suggesting the Sirt2/Foxo1 axis as a potential therapeutic target for ASD [30]. Restoration of Sirt2/Foxo1 expression by Rg1 indicates that its benefits may stem from preserving these key regulatory nodes, preventing the excessive oxidative and inflammatory cascades triggered by VPA.

Consistent with prior studies, VPA dramatically elevated proinflammatory cytokines (IL‐1β, IL‐6, TNF‐α) and MDA, whereas reducing SOD activity in both brain regions [17, 31, 32]. Neuroinflammation and oxidative stress form a vicious cycle in ASD models, compromising synaptic function and neuronal integrity [33]. By lowering these cytokines and restoring SOD levels, Rg1 ameliorates a key pathological axis in VPA‐induced toxicity. Its antioxidative and anti‐inflammatory properties have been reported in other neurological conditions [34, 35]. Here, the reduction of MDA and upregulation of SOD further confirm Rg1's capacity to modulate redox homeostasis—a critical step for maintaining neuronal health and sustaining behavioral recovery.

Despite the robust benefits observed, our study has several limitations. First, we employed only one ASD model (prenatal VPA exposure), which may limit the generalizability of our findings to other ASD subtypes or etiologies. Second, our investigation exclusively involved male mice, considering the higher prevalence of autism in males. Consequently, sex‐based differences and the efficacy of Rg1 in female subjects remain unexplored and warrant further investigation. Third, the lack of an Rg1‐only control group precludes definitive conclusions regarding the safety profile and specific therapeutic efficacy of Rg1 independent of VPA‐induced pathology; future studies should include this control condition to rigorously evaluate these aspects. Fourth, while we identified restoration of Sirt2/Foxo1 signaling and suppression of neuroinflammation and oxidative stress as critical mechanisms underlying Rg1's therapeutic effects, other potential contributors—including BDNF/TrkB or mTOR signaling pathways—were not examined and cannot be ruled out. Fifth, although we confirmed neuronal loss and partial rescue using Nissl staining, other histological markers of brain injury, such as neurogenesis (e.g., DCX) and glial responses (e.g., GFAP for astrocytosis and Iba1 for microgliosis), were not evaluated. As such, the extent of VPA‐induced neurodevelopmental pathology and the precise histological effects of Rg1 remain incompletely characterized and warrant further investigation. Sixth, the treatment duration in our study was relatively short, leaving it uncertain whether long‐term Rg1 administration would yield sustained behavioral and molecular improvements. Lastly, although existing literature suggests broader beneficial effects of Rg1 on neuronal development and neurotransmitter systems‐including modulation of dopaminergic and norepinephrinergic pathways relevant to ADHD pathology [36]‐our current findings specifically demonstrate therapeutic effects in a VPA‐induced neuronal developmental impairment model. Additional targeted studies using various models of neurodevelopmental disorders, such as ADHD, are required to clarify the broader therapeutic applicability and precise underlying mechanisms of Rg1.

In summary, our findings demonstrate that Rg1 counters VPA‐induced autism‐like behaviors—ranging from repetitive actions to impaired sociability—by modulating Sirt2/Foxo1 expression and reducing neuroinflammation and oxidative damage. These preclinical data support further inquiry into Rg1 as a promising candidate for ASD intervention.

## Conflicts of Interest

The authors declare no conflicts of interest.

## Supporting information


**Figure S1.** Ginsenoside Rg1 treatment does not affect body weight during the 29‐day experimental period. Data are shown as mean ± SD (*n* = 8 per group). Statistical analysis was conducted using two‐way ANOVA with Tukey’s multiple comparisons test. *indicate *p <* 0.05 compared with the Normal group.

## Data Availability

The data that support the findings of this study are available from the corresponding author upon reasonable request.
