# Supplementary figures and images for: Ginsenoside Rg1 Restores Sirt2/Foxo1 Expression and Alleviates Autism‐Like Behaviors in a Valproic Acid Induced Male Mouse Model
Source: Kaohsiung J Med Sci. 2025 Jul 7;41(11):e70078. doi: 10.1002/kjm2.70078 (PMC12622405; doi:10.1002/kjm2.70078)

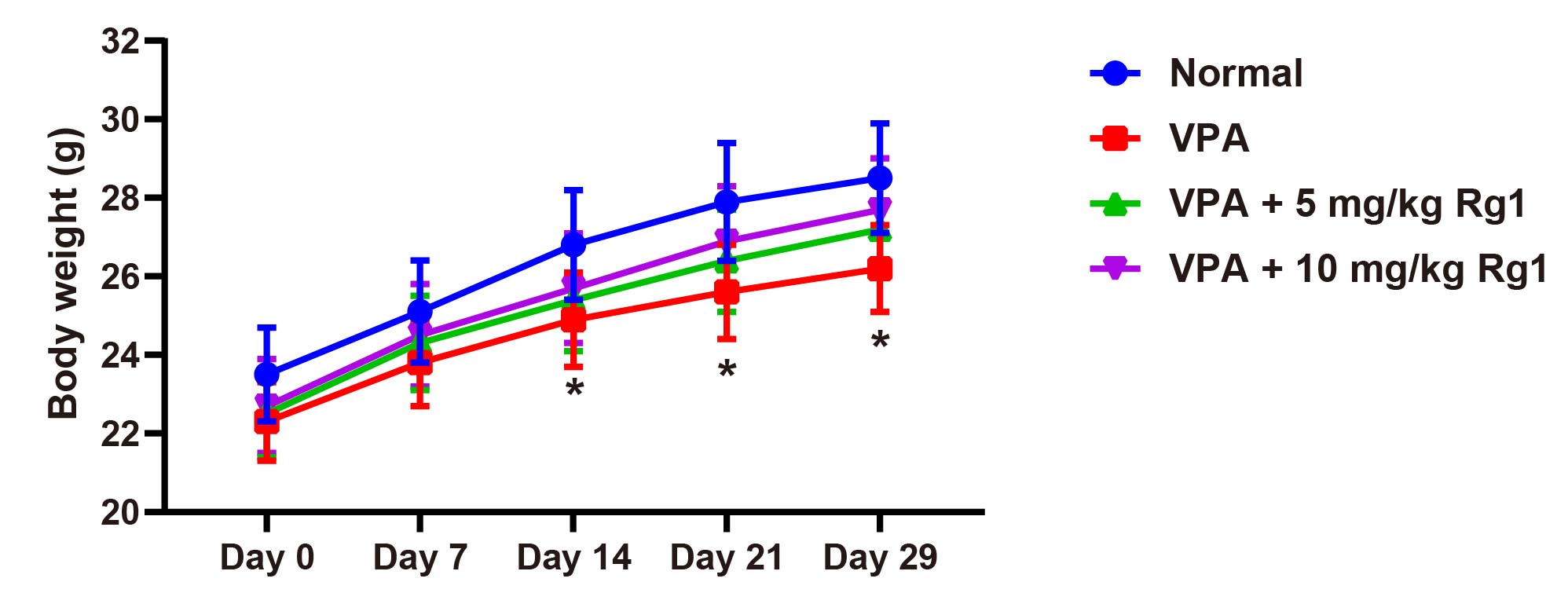

Supplement: Supplementary file 1 — Figure S1. Ginsenoside Rg1 treatment does not affect body weight during the 29‐day experimental period. Data are shown as mean ± SD (n = 8 per group). Statistical analysis was conducted using two‐way ANOVA with Tukey’s multiple comparisons test. *indicate p < 0.05 compared with the Normal group. [file KJM2-41-e70078-s001.jpg]
